# Factors Associated With Exposure to Antismoking Information Among Adults in Vietnam, Global Adult Tobacco Survey, 2010

**DOI:** 10.5888/pcd10.120348

**Published:** 2013-09-12

**Authors:** Kim Bao Giang, Hoang Van Minh, Pham Quynh Nga, Phan Thi Hai, Nguyen The Quan, Van T. Tong, Le Thi Thanh Xuan, Jason Hsia

**Affiliations:** Author Affiliations: Kim Bao Giang, Le Thi Thanh Xuan, Institute for Preventive Medicine and Public Health, Hanoi Medical University, Hanoi, Vietnam; Pham Quynh Nga, World Health Organization Office in Vietnam, Hanoi, Vietnam; Phan Thi Hai, Vietnam Steering Committee on Smoking and Health (VINACOSH), Hanoi, Vietnam; Nguyen The Quan, General Statistics Office, Hanoi, Vietnam; Van T. Tong, Jason Hsia, Centers for Disease Control and Prevention, Atlanta, Georgia.

## Abstract

**Introduction:**

The media play a critical role in tobacco control. Knowledge about the exposure of a population to antismoking information can provide information for planning communication activities in tobacco control. We examined exposure to antismoking information associated with socioeconomic and demographic factors among adults (≥15 years) in Vietnam.

**Methods:**

The Global Adult Tobacco Survey (GATS) is a nationally representative household survey of noninstitutionalized men and women aged 15 years or older and was conducted in Vietnam in 2010 (N = 9,925). We used GATS data on exposure to sources of antismoking information and analyzed associations among socioeconomic and demographic groups.

**Results:**

An estimated 91.6% of the adult population was exposed to at least 1 source of antismoking information, and the mean number of sources of exposure was 3.7. Compared with their counterparts, respondents who were older, had higher education levels, higher economic status, and higher knowledge levels about the health consequences of smoking were more likely to be exposed to any source of antismoking information and to more informational sources. The most common source of exposure was television (85.9%). Respondents of higher social class (education, occupation, wealth) had more exposure through modern media sources (television), and respondents of lower social class were exposed to more traditional sources such as radio or loudspeakers.

**Conclusion:**

Exposure to at least 1 source of antismoking information is high in Vietnam, and the number and type of source varied by sociodemographic group. Use of multiple communication channels is recommended to reinforce antismoking messages and to reach different groups in the population.

## Introduction

Mass communication plays a critical role in tobacco control (1–3); communication channels include newspapers, magazines, radio, and television. Effective mass media campaigns are a key part of a tobacco control strategy because they can help prevent young people from starting to smoke, encourage smokers to stop or not to smoke around nonsmokers, and change the social context of tobacco use (4).Conducting antitobacco media campaigns combined with school-based and community interventions is an effective approach for preventing initiation of smoking and increasing rates of smoking cessation (5). Similarly, antitobacco media campaigns can reduce the sales of cigarettes (6). Thus, exposure of the population to antismoking information is important to encourage cessation, to reduce the consumption and use of tobacco, and to help keep teenagers and other young people from initiating smoking (1,2).

Vietnam has a high smoking prevalence among men and a low smoking prevalence among women. In 2002, prevalence was 56.1% among men and 1.8% among women (7); in 2010, it was 47.4% among men and 1.4% among women (8). Although questionnaire, sampling, and data collection methods of the surveys in 2010 differed from the survey in 2002, the slightly lower smoking prevalence in 2010 shows that reduction of smoking prevalence was small.

Vietnam signed the World Health Organization Framework Convention on Tobacco Control (FCTC) on August 8, 2003, and ratified it on November 17, 2004. The FCTC, the first international public health treaty, was developed in response to the globalization of the tobacco use epidemic and is an evidence-based treaty that reaffirms the right of all people to the highest standard of health. Article 12 of FCTC requires the signing parties to promote and strengthen public awareness of tobacco control issues, using all available and appropriate communication tools and providing broad access to effective and comprehensive educational and public information on the health risks of tobacco use, including the addictive characteristics of tobacco (9). During the past 10 years, several tobacco control measures have been conducted in Vietnam, including banning tobacco advertising, increasing the tobacco tax, banning tobacco smoking inside public places and workplaces, implementing textual health warnings on cigarette packages, and disseminating antismoking communications on the negative health effects of smoking on television, on radio, and via other communication means.

In Vietnam, health education and communication are among the important tobacco control strategies (10,11). However, little is known about the exposure of different groups to antismoking sources. Evidence of exposure to different antismoking information sources by various socioeconomic groups could be used to evaluate and inform national tobacco control activities. This article aims to describe and examine associations of exposure to antismoking information in mass media with socioeconomic and demographic characteristics of adults (≥15 years) in Vietnam.

## Methods

We analyzed data from the Global Adult Tobacco Survey (GATS) conducted in Vietnam in 2010 (8). Following a standardized protocol, the GATS in Vietnam is a nationally representative survey of households with noninstitutionalized adults aged 15 years or older (12).

The standard GATS questionnaire collects data on adult tobacco use and key measures of tobacco control. The questionnaire was translated and adapted to the Vietnamese context.

Data were collected by 26 teams, each consisting of 1 team leader and 4 interviewers to ensure close supervision and the collection of high-quality data. Data collection was conducted using an iPAQ (Hewlett Packard, Palo Alto, California), a portable handheld device, instead of using a traditional paper-based survey. Before conducting the field work, a real-time case file containing addresses and names of the households assigned to the interviewer was preloaded in the iPAQ; the interviews were conducted in Vietnamese. All the responses were entered into the iPAQ. Data were collected from March 22, through May 13, 2010, in all 63 provinces of Vietnam.

The dependent variable was “exposure to antismoking information during the last 30 days prior to the interview” from newspapers and magazines, television, radio, billboards, the Internet, local radio or a loudspeaker, posters, and leaflets or pamphlets. Antismoking information could be any information related to the health effects of tobacco smoking, disease and socioeconomic burden caused by smoking, or smoking cessation methods that were seen or heard from different media. Independent variables were sex, age in years (15-24, 25-44, 45-64, or ≥65), educational level (primary or less, lower secondary, upper secondary, college and/or university degree), occupation (manager or professional, office worker, service or sales, farming, forestry or fishing, construction or mining, production or machine, or other), asset-based wealth quintile (from the poorest to the richest in increments of 20%), residence (urban or rural), smoking status, and knowledge of the health consequences of smoking (don’t know; know the 3 main health consequences [lung cancer, stroke, heart disease] of active smoking or passive smoking; or know the 3 main health consequences of both active and passive smoking).

Both descriptive and analytical statistical analyses were conducted by using Stata 10 (StataCorp, LP, College Station, Texas) software. Percentages and frequencies of exposure to antismoking information and, where appropriate, odds ratios and their corresponding 95% confidence intervals (CIs), were calculated by demographic variable. Multivariate logistic regression modeling was performed to examine the association between exposure to antismoking information and sociodemographic variables (including smoking status). Linear regression was used to examine the association between the number of sources of antismoking information and sociodemographic variables. In the first model of logistic regression and linear regression, education level was included and respondents younger than 25 were excluded because of the assumption that the educational levels of these respondents are not stable. All data were weighted to be representative of households with noninstitutionalized adults aged 15 years or older in Vietnam. A significance level of .05 was used.

## Results

The 9,925 completed interviews made up a response rate of 92.7% (93.9% in rural areas and 91.7% in urban sites). The interviews represented an estimated 64.3 million adults aged 15 years or older in Vietnam (Table 1); 51.4% of the study population was female. People aged 25 to 44 years made up the largest weighted proportion (41.9%), and those aged 65 or older accounted for the smallest share (8.8%). Most of the study population had either a lower secondary education (52.5%) or primary or less education (26.0%). The most common occupation in the study population was farming (49.6%). More than two-thirds of people aged 15 or older lived in rural areas.

**Table 1:** Distribution of Study Subjects, by Selected Sociodemographic Characteristics, Global Adult Tobacco Survey, Vietnam, 2010

Characteristics	Sample Size^a^	Weighted % (95% Confidence Interval)
**Sex**		
Male	4,356	48.6 (47.3–49.9)
Female	5,569	51.4 (50.1–52.7)
**Age**		
15–24	1,656	25.9 (24.6–27.2)
25–44	4,251	41.9 (40.6–43.2)
45–64	2,886	23.4 (22.4–24.5)
≥65	1,132	8.8 (8.2–9.5)
**Education**		
Primary or less	2,034	26.0 (24.2–27.8)
Lower secondary	3,981	52.5 (50.8–54.3)
Upper secondary	1,023	14.3 (13.1–15.5)
College and/or a university degree	1,227	7.2 (6.6–7.9)
**Occupation**		
Manager or professional	845	6.6 (5.9–7.5)
Office worker	220	2.0 (1.6–2.3)
Service or sales	1,589	19.2 (17.8–20.6)
Farming	3,069	49.6 (47.3–51.8)
Forestry or fishing	120	1.8 (1.3–2.6)
Construction or mining	317	5.2 (4.5–6.0)
Production or machine	834	12.9 (11.7–14.3)
Other	248	2.7 (2.3–3.3)
**Residence**		
Urban	4,958	30.7 (30.0–31.4)
Rural	4,967	69.3 (68.6–70.0)
**Total**	9,925	100

a Not all values sum to total because not all participants were included in some categories.

The most common channel of antismoking information for Vietnamese adults was television (85.9%); Internet (12.0%) and leaflets or pamphlets (7.7%) were the least common channels. Overall, exposure to antismoking information from at least 1 channel was 91.6% (Figure), and mean number of channels of antismoking information was 3.7 (95% confidence interval [CI], 3.67–3.76) (Table 2).

**Figure:**
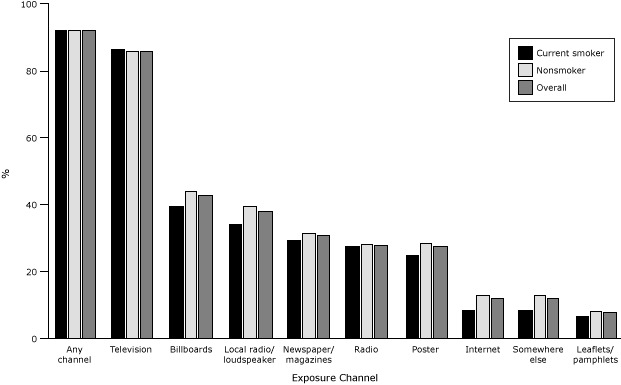
Exposure to antismoking information, by exposure channel and smoking status, Vietnam, 2010. **Table:** 

Exposure Channel	Current Smoker	Nonsmoker	Overall
	%		
Any channel	91.6	91.6	91.6
Television	86.4	85.8	85.9
Billboards	39.3	43.9	42.8
Local radio/loudspeaker	34.2	39.4	38.2
Newspaper/magazines	29.5	31.2	30.8
Radio	27.7	28.1	28.0
Poster	24.8	28.5	27.6
Internet	8.3	13.1	12.0
Somewhere else	8.3	13.1	12.0
Leaflets/pamphlets	6.4	8.0	7.7

**Table 2:** Linear Regression Analysis of the Association of Mean Number of Antismoking Information Channels and Selected Sociodemographic Factors, Global Adult Tobacco Survey, Vietnam, 2010

Variables	Mean No. of Antismoking Information Channels	Model 1^a^ (Education Included, Aged ≥25)		Model 2^b^ (Education Excluded, All Ages)	
		*F*	*P* Value	*F*	*P* Value
**Sex**					
Male	3.9	1 [Reference]	.25	1 [Reference]	.66
Female	3.7	−0.09		−0.03	
**Age, y**					
15–24	4.1	NA	NA	1 [Reference]	
25–44	3.8	1 [Reference]	NA	0.13	.20
45–64	3.9	0.09	.12	0.17	.12
≥65	3.1	0.13	.27	0.05	.74
**Education**					
Primary or less	2.6	1 [Reference]	NA	NA	NA
Lower secondary	3.7	0.49	<.001		
Upper secondary	4.4	0.66	<.001		
College and/or a university degree	5.0	0.93	<.001		
**Occupation**					
Manager/professional	5.1	1 [Reference]	NA	1 [Reference]	NA
Office worker	5.2	0.14	.53	0.05	.82
Service or sales	3.9	−0.42	.006	−0.72	<.001
Farming	3.2	−0.42	.009	−0.81	<.001
Forestry or fishing	3.3	−0.48	.06	−0.94	<.001
Construction or mining	3.2	−0.35	.08	−0.88	<.001
Production or machine	3.7	−0.35	.03	−0.71	<.001
Other	4.3	−0.06	.77	−0.40	<.001
**Residence**					
Urban	4.2	1 [Reference]	.14	1 [Reference]	.02
Rural	3.5	−0.10		−0.17	
**Smoking status**					
Smoker	2.2	1 [Reference]	.26	1 [Reference]	.91
Nonsmoker	3.8	0.09		−0.01	
**Wealth quintile**					
1 (poorest)	2.7	1 [Reference]	NA	1 [Reference]	NA
2	3.5	0.54	<.001	0.55	<.001
3	3.7	0.55	<.001	0.67	<.001
4	4.0	0.83	<.001	0.98	<.001
5 (richest)	4.7	1.22	<.001	1.38	<.001
**Knowledge of health consequences of smoking**					
Don’t know	2.8	1 [Reference]	NA	1 [Reference]	NA
Knowledge about 3 main consequences^c^ of active or passive smoking	3.7	0.67	<.001	0.76	<.001
Knowledge about 3 main consequences^c^ of both active and passive smoking	4.4	1.33	<.001	1.41	<.001

Abbreviation: NA, not applicable.

a Model 1 is significant with an intercept of 2.53, and all variables in this model explain 19.6% of the variation in number of antismoking information sources.

b Model 2 is significant with an intercept of 3.21, and all variables in this model explain 18.7% of the variation in number of antismoking information sources.

c Lung cancer, stroke, or heart disease.

 Smokers did not differ from nonsmokers in the exposure to at least 1 antismoking information channel. Compared with current smokers, nonsmokers had slightly higher rates of exposure to information on billboards (43.9% vs 39.3%), the Internet (13.1% vs 8.3%), local radio or a loudspeaker (39.4% vs 34.2%), and posters (28.5% vs 24.8%) (Figure); these differences were significant. Linear regression and logistic regressions models, however, did not indicate any differences between these 2 groups with regard to number of antismoking information channels and exposure to any channels (Tables 2 and 3).

**Table 3:** Logistic Regression Analysis of the Association Between Having Any Exposure to Antismoking Information and Selected Sociodemographic Factors, Global Adult Tobacco Survey, Vietnam, 2010

Variable	Model 1 (Education Included, Aged ≥25), OR (95% CI)	Model 2 (Education Excluded, All Ages), OR (95% CI)
**Sex**		
Male	1 [Reference]	
Female	0.9 (0.6–1.2)	0.9 (0.7–1.3)
**Age, y**		
15–24	NA	
25–44	1 [Reference]	1.5 (1.1–2.1)
45–64	1.4 (1.1–1.9)	2.0 (1.3–2.9)
≥65	1.5 (0.9–2.5)	1.5 (0.9–2.7)
**Education**		
Primary or less	1 [Reference]	NA
Lower secondary	2.4 (1.9–3.1)	
Upper secondary	3.9 (2.3–6.7)	
College and/or a university degree	2.5 (1.1–5.5)	
**Occupation**		
Manager or professional	1 [Reference]	1 [Reference]
Office worker	3.6 (0.6–23.0)	1.9 (0.5–7.8)
Service or sales	1.0 (0.4–2.6)	0.8 (0.4–1.5)
Farming	1.1 (0.4–3.1)	0.9 (0.5–1.6)
Forestry or fishing	1.4 (0.4–5.3)	0.8 (0.3–2.1)
Construction or mining	1.3 (0.4–4.2)	0.5 (0.2–1.2)
Production or machine	0.9 (0.3–2.4)	0.9 (0.4–1.7)
Other	1.0 (0.4–2.8)	1.3 (0.6–2.8)
**Residence**		
Urban	1 [Reference]	1 [Reference]
Rural	1.0 (0.8–1.3)	0.9 (0.7–1.2)
**Smoking status**		
Smoker	1 [Reference]	1 [Reference]
Nonsmoker	0.9 (0.6–1.3)	0.8 (0.6–1.2)
**Wealth quintile**		
1 (poorest)	1 [Reference]	1 [Reference]
2	2.5 (1.8–3.5)	2.6 (1.9–3.6)
3	2.4 (1.5–3.6)	3.1 (2.0–4.7)
4	2.3 (1.6–3.5)	3.5 (2.4–5.1)
5 (richest)	2.8 (1.8–4.4)	4.1 (2.6–6.4)
**Knowledge of health consequences of smoking**		
Don’t know	1 [Reference]	1 [Reference]
Knowledge about 3 main consequences^a^ of active or passive smoking	2.6 (1.9–3.4)	2.9 (2.2–3.7)
Knowledge about 3 main consequences^a^ of both active and passive smoking	5.6 (4.0–7.7)	4.7 (3.4–6.5)

Abbreviations: OR, odds ratio; CI, confidence interval; NA, not applicable.

a Lung cancer, stroke, or heart disease.

Stratified analyses on exposure to antismoking information (data not shown) were conducted by age group, residence, educational attainment, occupation, and level of wealth. Only differences between sexes were found. Men had a significantly higher rate of exposure to the Internet (13.4% vs 10.7%), while women had a significantly higher rate of exposure to local radio or a loudspeaker (42.1% vs 34.1%). According to linear regression and logistic regression analyses, men did not differ from women in the exposure to communication channels nor in number of information channels accessed (Tables 2 and 3).

Exposure to antismoking information was significantly higher among respondents from urban areas than those from rural areas from newspapers or magazines (42.2% vs 25.7%), billboards (53.8% vs 37.9%), the Internet (21.8% vs 7.6%), and posters (41.1% vs 21.7%). In contrast, respondents from rural areas had a significantly higher rate of exposure antismoking information from local radio or loudspeakers (40.3% vs 33.4%) and nonlocal radio (29.5% vs 24.7%) than did their urban counterparts. Linear regression and logistic regression models did not find any differences between those from urban and rural areas with regard to number of information sources accessed and exposure to communication channels (Table 2 and 3).

Results of linear regression analysis did not indicate any differences between age groups (Table 2). Compared with respondents aged 15 to 24, respondents aged 45 to 64 were 1.4 (model 1) or 2.0 (model 2) times as likely to be exposed to antismoking information. In model 2, respondents aged 25 to 44 were 1.5 times as likely as respondents aged 15 to 24 to be exposed to antismoking information (Table 3).

Respondents with higher education levels were more likely than those with less education to be exposed to antismoking information from each of the channels listed in the Figure, except for exposure from local radio or loudspeaker. Linear regression model 1 indicated that higher education was a significant predictor for exposure to more antismoking information channels (Table 2) and that respondents with higher education levels were 2.4 to 3.9 times as likely to be exposed to antismoking information compared with respondents with a primary or less education (Table 3).

Respondents from the richest group had more exposure than the poorest group to antismoking information in newspapers or magazines, on billboards, on the Internet, and through posters and leaflets or pamphlets, while the poorest group had slightly more exposure than the richest group to local radio or loudspeakers (35.8% vs 32.3%). Linear regression model 1 found that respondents from the wealthiest income groups were exposed to more antismoking information channels than those from the poorest income groups (Table 2). Both logistic regression models found that wealth quintiles 2 through 5 of the population were 2.3 to 4.1 times as likely to be exposed to antismoking information than the poorest group (quintile 1).

Better knowledge about the health consequences of smoking was associated with exposure to more informational channels (Table 2). Model 1 indicated that respondents with knowledge about health consequences of active or passive smoking were 2.6 times (95% CI, 1.9–3.4) as likely to be exposed to antismoking information as those with no knowledge, and those with knowledge about health consequences of both active and passive smoking were 5.6 times (95% CI, 4.0–7.7) as likely to be exposed as those with no knowledge. In model 2 the comparable odds were 2.9 (95% CI, 2.2–3.7) and 4.7 (95% CI, 3.4–6.5).

## Discussion

We found that 91.6% of the adult population in Vietnam self-reported that they were exposed to at least 1 antismoking information channel in the previous 30 days. These data suggest that tobacco control efforts have been successful at penetrating the general adult population and that people remember these messages. However, more work is needed to reach subpopulations that were least likely to report having been exposed to antismoking information, specifically those who were younger, had a lower education, or were of lower economic status. This prevalence of exposure is higher than that reported by GATS for the Philippines (80.8%) (13), Thailand (86.9%) (14), or Bangladesh (49.8%) (15). We found that the mean number of antismoking information channels for adults was 3.7. Evidence from health communication research has found that the higher level of exposure to health communication messages via different modalities is associated with increases in positive changes in knowledge and attitudes for behavior change (16,17). The high level exposure to antismoking messages in Vietnam can be seen as a positive outcome of the several efforts made by tobacco control agencies in this country during the past decade.

Among all communication channels, most respondents reported being exposed to antismoking information through television (85.9%). Most households in Vietnam now have access to a television (100% among households in urban areas and 85.7% among rural households), and this medium is an important source of information in this country (18). Antismoking information on the Internet (12%) was viewed far less commonly by the adult population than was antismoking information via television, as in 2010 only 27% of the Vietnamese population had access to it (19). However, Internet use is becoming more prevalent in Vietnam; the number of people using it increases approximately 10% each year (20,21). In our study, we found that younger adults were more likely than older adults to use the Internet. The Internet should be considered in future efforts to communicate information about tobacco control to the Vietnamese population.

In our study, exposure to antismoking information by communication channels differed by sociodemographic groups. We found that respondents who were male, of urban residence, more educated, and of higher socioeconomic status were more likely to be exposed to antismoking messages through the more “modern” communication channels (eg, television) than the more “traditional” channels (eg, loudspeakers, radio). These findings provide good support for selecting communication channels based on sociodemographic characteristics.

We found that having exposure to any media sources did not differ by smoking status, but the mean number of antismoking information channels was higher, though not significant, in nonsmokers than in smokers (3.8 vs 2.2). Nevertheless, this finding is consistent with results from GATS in the Philippines (13) and in Thailand (14).

Finally, our results indicated a significant association between exposure to antismoking information and knowledge of the health consequences of smoking, although we are not able to assess causation. Each communication channel has its own strengths and weaknesses, but conveying messages through several channels would make it easier for recipients to be reached and would make the messages more convincing and easier to remember. Thus, those who have exposure to more information sources are more likely to have better knowledge about smoking, and this knowledge would likely result in behaviors such as not to start smoking or to quit smoking.

The high level of exposure to antismoking information channels does not ensure that current communication programs in tobacco control are effective. Effectiveness of a communication program depends on several factors, including communication contents, methods, frequencies, capacity of information sources, and characteristics of the audience. One limitation to our study was that we could not determine the contents of antismoking information, which may have affected recipients’ knowledge and smoking behaviors differently because the information may have varied within and between information sources. However, evidence from our study can be used to plan communication programs aiming to reach different groups in the society.

We found that the prevalence of being exposed to at least 1 source of antismoking information was high among adults in Vietnam. However, exposure differed across sociodemographic groups. Respondents of higher social class often had more exposure to different sources of antismoking information and via more modern media sources, while respondents of lower social class were exposed to more traditional sources such as radio or loudspeakers. Use of multiple communication channels is recommended to reinforce antismoking messages and to reach different groups in the population. To maintain the achievements of the communication campaign in Vietnam and to improve the effectiveness of tobacco control activities, strong and continuous antismoking communication programs are necessary.

## References

[1] National Cancer Institute. The role of the media in promoting and reducing tobacco use. Tobacco Control Monograph no. 19 (NIH Pub. No. 07-6242). Bethesda (MD): US Department of Health and Human Services, National Institutes of Health, National Cancer Institute; 2008.

[2] WHO Report on the global tobacco epidemic, 2009: the MPOWER packaged. Geneva (CH): World Health Organization; 2009.

[3] Regional action plan for the tobacco free initiative in the western Pacific region (2010–2014). Manila (PH): World Health Organization; 2010.

[4] Increasing access to effective anti-tobacco mass media campaigns. J Cancer Institute. 2009 Dec;(2).

[5] Comprehensive tobacco prevention and cessation programs effectively reduce tobacco use. Washington (DC): Campaign for Tobacco-Free Kids; 2013.

[6] Hu TW , Sung HY , Keeler TE . Reducing cigarette consumption in California: tobacco taxes vs an anti-smoking media campaign. Am J Public Health 1995;85(9):1218–22. 10.2105/AJPH.85.9.1218 7661228 PMC1615589

[7] National Health Survey 2001–2002. Hanoi (VN): Ministry of Health, General Statistics Office of Vietnam, Medical Publishing House; 2003.

[8] Global Adult Tobacco Survey Vietnam 2010. Hanoi (VN): Ministry of Health of Vietnam, Hanoi Medical University, General Statistics Office of Vietnam, Centers for Disease Control and Prevention, World Health Organization; 2010.

[9] WHO framework convention on tobacco control. Geneva (CH): World Health Organization; 2003.

[10] National policy on tobacco control and prevention in the period 2000–2010. Resolution no. 12/2000/NQ-CP. August 14, 2000.

[11] Action plan for the implementation of the WHO framework convention on tobacco control. Prime Minister Decision no. 1315/QĐ-TTg; 2009.

[12] Global Adult Tobacco Survey (GATS). Sample design manual. Geneva (CH): World Health Organization; 2008.

[13] Philippines Global Adult Tobacco Survey: country report 2010. Geneva (CH): World Health Organization, Centers for Disease Control and Prevention, Philippines National Statistics Office, Philippines Department of Health; 2010.

[14] Global Adult Tobacco Survey: Thailand report 2010. Geneva (CH): World Health Organization, Centers for Disease Control and Prevention, Thailand Department of Health; 2010.

[15] Global Adult Tobacco Survey: Bangladesh report 2009. Geneva (CH): World Health Organization, Centers for Disease Control and Prevention; 2009.

[16] Piotrow PT , Kincaid DL , Rimon JG II, Rinehart W . Health communication: lessons from family planning and reproductive health. Westport (CT): Praeger; 1997.

[17] Reinert B , Carver V , Range L . Anti-tobacco messages from different sources make a difference with secondary school students. J Public Health Manag Pract 2004;10(6):518–23. 15643375 10.1097/00124784-200411000-00008

[18] Results of the household living standard 2008. Hanoi (VN): General Statistics Office of Vietnam, Statistical Publishing House; 2009.

[19] Internet world statistics: usage and population statistics. Vietnam Internet Usage Stats and Marketing Report; 2010.

[20] Socioeconomic situation of Vietnam in 2009. Hanoi (VN): General Statistics Office of Vietnam; 2009.

[21] General Statistics Office of Vietnam. Socioeconomic situation of Vietnam in November 2010. Hanoi (VN): General Statistics Office of Vietnam; 2010.

